# Drug addiction: from bench to bedside

**DOI:** 10.1038/s41398-021-01542-0

**Published:** 2021-08-12

**Authors:** Julian Cheron, Alban de Kerchove d’Exaerde

**Affiliations:** Laboratory of Neurophysiology, ULB Neuroscience Institute, Université Libre de Bruxelles (ULB), Brussels, B-1070 Belgium

**Keywords:** Molecular neuroscience, Addiction

## Abstract

Drug addiction is responsible for millions of deaths per year around the world. Still, its management as a chronic disease is shadowed by misconceptions from the general public. Indeed, drug consumers are often labelled as “weak”, “immoral” or “depraved”. Consequently, drug addiction is often perceived as an individual problem and not societal. In technical terms, drug addiction is defined as a chronic, relapsing disease resulting from sustained effects of drugs on the brain. Through a better characterisation of the cerebral circuits involved, and the long-term modifications of the brain induced by addictive drugs administrations, first, we might be able to change the way the general public see the patient who is suffering from drug addiction, and second, we might be able to find new treatments to normalise the altered brain homeostasis. In this review, we synthetise the contribution of fundamental research to the understanding drug addiction and its contribution to potential novel therapeutics. Mostly based on drug-induced modifications of synaptic plasticity and epigenetic mechanisms (and their behavioural correlates) and after demonstration of their reversibility, we tried to highlight promising therapeutics. We also underline the specific temporal dynamics and psychosocial aspects of this complex psychiatric disease adding parameters to be considered in clinical trials and paving the way to test new therapeutic venues.

## Introduction

Drug addiction including smoking, alcohol and illicit drug use is indirectly or directly responsible for 11.8 million deaths each year in the world [1]. According to the Global Burden of Disease study, this number is higher than deaths from cancer and accounts for a fifth of all deaths around the world [1].

Drug addiction is defined as a chronic, relapsing disease that results from the prolonged effects of drugs on the brain. Similarly to other neuropsychiatric diseases, drug addiction is intermingled with behavioural and social aspects that are equally important parts of the disease, complicating the overall therapeutic approach. Actually, it is only recently, in the beginning of the 21st century, that we started to see “the drug-addict” as someone suffering from a disease and whose brain has been altered fundamentally by drugs [2]. Therefore, the most effective treatment approaches include biological, behavioural and social-context components. Based on the latest scientific advances, treatment and management of drug addiction patients point towards a personalised strategy [3]. However, there are very few objective and effective strategies for treating drug addiction. Without the mandatory mechanistic basic knowledge on drug addiction, the development of new therapeutic strategies is postponed.

The neurobiological circuits and mechanisms that support compulsive seeking and consumption of drugs with addictive potential are partially known. They comprise a progressive shift in the involvement of ventral to dorsal and medial to lateral striatal circuitry [4, 5], along with molecular and cellular adaptations to drugs of abuse exposure. They include neuronal and synaptic plasticity and modifications in gene expression, in part through epigenetic mechanisms [6]. Notably, drug-induced neuronal modifications can also occur in non-pathological processes, underlying the fact that drugs of abuse hijack normal adaptive changes in the brain [7]. Indeed, laboratory and clinical observations suggest that addiction is driven by the usurpation of neuronal processes that normally serve reward-related learning and memory. Most of the modifications that have been shown to be involved in a state of addiction (modified gene transcription, epigenetics, neuronal plasticity and neurotrophic mechanisms) are also associated with physiological forms of behavioural memory in murine model such as spatial memory, fear conditioning and operant conditioning [7, 8].

We know that only a proportion of individuals (depending of the drug type) will develop drug addiction after several exposures [9]. This individual vulnerability is probably linked to both genetic and environmental factors [10]. Drug addiction is highly polygenic, as hundreds of genetic variations combined result in variable vulnerability [11, 12]. Several types of environmental factors have been characterised and interact with an individual genetic background [12, 13]. Psychosocial stress is one of the factors, but the most important one, is by far, the exposure to drugs of abuse. Usually, drug abuse starts with a ‘gateway’ drug (mostly socially driven) catapulting the individual vulnerability to other drugs of abuse [14].

During the last three decades, combine effort has been dedicated to identify brain regions and molecular pathways involved in the development of addiction to drugs of abuse. Here, we will focus on experimental approaches that helped to provide a clearer picture on the physiopathology of drug addiction guiding therapeutic opportunities.

## Converging actions on brain reward pathway elicit its remodelling

**Fig. 1:**
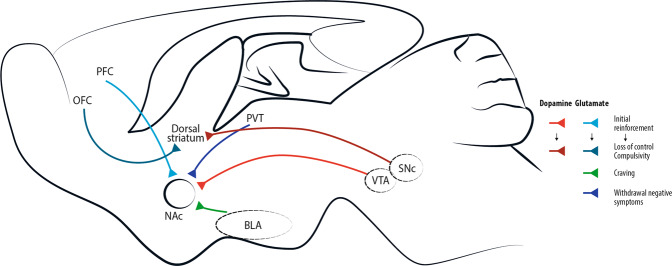
Simplified brain circuits implicated in drug addiction. Addictive drugs of different types have a common effect of increasing levels of dopamine released by neurons projecting from the ventral tegmental area (VTA). This effect is central for initial drug reinforcement. Notably, drug taking with initial reinforcement involves a potentiation of the projection from prefrontal cortex (PFC) to nucleus accumbens (NAc), while other glutamatergic projections are mostly involved in craving, like basolateral amygdala (BLA)-NAc projection, or in withdrawal/negative symptoms, like paraventricular thalamus (PVT)-NAc projection. With increasing administration of drugs of abuse and progressive shift toward compulsive abuse, the dorsal (dorso-lateral) striatum seems more and more implicated, with dopaminergic cells involved shifting progressively from the VTA to the substantia nigra pars compacta (SNc) [4]. Recently, data acquired through optogenetic dopamine neuron self-stimulation suggested prominent synaptic strengthening of the orbitofrontal cortex (OFC) to dorsal (dorso-medial) striatum projection in compulsive mice [31].

The circuit at the centre of the disease is the mesolimbic pathway, also referred as the reward pathway (Fig. 1). The mesolimbic pathway includes dopaminergic neurons in the ventral tegmental area (VTA) of the midbrain and their targets in the limbic forebrain, especially the nucleus accumbens (NAc), a major component of the ventral striatum. The GABA medium-sized spiny neurons (MSNs, ~95% of striatal neurons), which are targets of glutamatergic and dopaminergic inputs, form two pathways [15]. The dopamine D1 receptor–positive (D1R) striatonigral MSNs project to the medial globus pallidus and substantia nigra pars reticulata (direct pathway) and coexpress dopamine D1 receptors and substance P, whereas D2R striatopallidal MSNs project to the lateral globus pallidus (indirect pathway) and coexpress dopamine D2 receptor, adenosine A2A receptor and enkephalin [16, 17]. Through different initial mechanisms, drugs of abuse increase the release of dopamine in the NAc from the VTA [18–20]. This VTA-NAc pathway could be seen as *primum movens* for the acute rewarding effects of all drugs of abuse. Regardless that drugs of abuse have distinct protein targets and mechanisms of action, in the end, the main addiction-related modifications are common to nearly all drugs of abuse and converge on the VTA and NAc with common acute functional effects [21]. It is schematically conjectured, that when stimulated by dopamine, cells in the NAc produce feelings of reward and satisfaction [22]. The physiological function of this response is to facilitate the motivation for basic biological goal-directed behaviours as survival, social interaction and reproduction. By artificially causing a build-up of dopamine in the NAc, drugs of abuse generate an artificial reward effect [22]. As all drugs of abuse increase dopaminergic transmission to the NAc after acute administration, they also produce shared modifications in the mesolimbic system after chronic exposure. They include (i) hypofunction of the dopamine pathway that is seen as a major contributor to the negative emotional symptoms associated to drug withdrawal, leading to drug craving and relapse, and (ii) drug-induced adaptations in glutamatergic afferents to the NAc [23, 24]. Clearly, these modifications in the mesolimbic system after the exposure to drugs of abuse is oversimplified. The hypofunction of dopaminergic system hypothesis is self-fulfilling in that research work has principally focused on dopamine to the exclusion of other neurotransmitters. Actually, some drug of abuse reinforcement appears to be independent of the mesocorticolimbic dopamine system (e.g. opioids [25], nicotine [26]), but support self-administration by imitating the effect of dopamine in the nucleus accumbens [21, 27, 28].

**Fig. 2:**
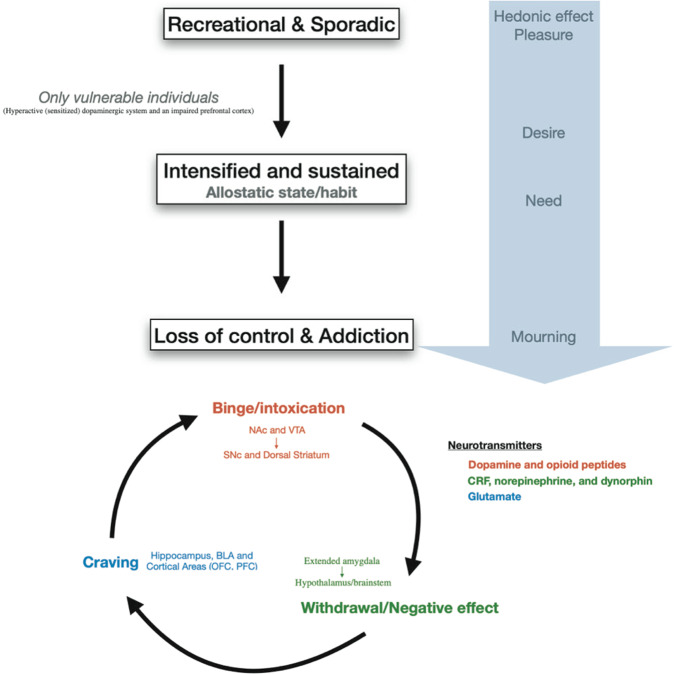
Progression to addiction and its three main stages. Progression to addiction is defined as a transition between three consecutive phases [252]: (1) Recreational, sporadic drug taking, in which drug of abuse administration is occasional and one activity among many other distractions of the individual. (2) Intensified and sustained drug use, in which drug administration strengthens and becomes the principal recreational activity of the individual; at this phase drug taking becomes a habit. (3) Loss of control of drug use and addiction, in which drug seeking and taking are now the principal activity of the patient. The first phase can occur to every person as drugs of abuse hijack the same brain circuit as natural rewards. The second phase occurs only in vulnerable users. The phase of addiction seems to be due to a second vulnerable trait with loss of control and compulsivity. Three stages of addiction are described [27]: (1) Binge/intoxication stage: reinforcing effects of drugs may initially use mainly dopamine and opioid peptides in the nucleus accumbens (NAc) and involves the ventral tegmental area (VTA). Subsequently, cue–response habits develop and includes the substantia nigra pars compacta (SNc) and the dorsal striatum. (2) Withdrawal/negative affect stage: the negative emotional state of withdrawal may involve the extended amygdala with corticotropin-releasing factor (CRF), norepinephrine and dynorphin as key neurotransmitters. Main projections of the extended amygdala consist of the hypothalamus and brainstem. (3) Craving stage: this stage includes conditioned reinforcement in the basolateral amygdala (BLA) and contextual processes in the hippocampus. This is controlled by cortical areas (prefrontal cortex (PFC) and orbitofrontal cortex (OFC)). A key neurotransmitter involved in the craving stage is glutamate.

Drug addiction is conceptually defined as a three-stages cycle: (1) consumption/binge/intoxication, (2) withdrawal with its negative affect and (3) craving stage (Fig. 2) [27]. Animal models and human imaging studies have exposed the different brain areas involved in each of these stages. Briefly, the VTA-NAc (for reinforcement) and dorsal striatum (for stimulus-response habits) are important for the consumption/binge/intoxication stage, the extended amygdala with the hypothalamus and the brainstem in the withdrawal stage and cortical areas, the dorsal striatum, the hippocampus and the basolateral amygdala in the craving stage (Fig. 2).

The progression of drug addiction begins with the first exposure, mostly when the drug is taken voluntarily for its recreational and hedonic effect, and progressively consolidates during repeated but still controlled drug use. While administration intensifies along with loss of control over drug intake, drug use becomes habitual and compulsive in vulnerable individuals [4, 29, 30] (Fig. 2). This progression from voluntary drug intake to habitual and compulsive use represents a progression from ventromedial to more dorsolateral regions of the striatum and from prefrontal cortex (PFC) to orbitofrontal (OFC) and more global cortical region [4, 31] (Fig. 1).

### Synaptic plasticity

Brain plasticity is a fascinating capacity allowing appropriate modification of the neural activity in response to new experiences and environmental stimuli [32]. Modifying the synaptic strength between neurons is widely assumed to be the mechanism by which memory is encoded and stored in the brain [7]. Hence, it is appealing to hypothesise that drugs of abuse cause long-term alterations on behaviour by changing synaptic plasticity in key brain circuits [4, 7, 32].

Drugs of abuse such as cocaine induce specific synaptic plasticity in the mesolimbic circuitry. One single injection of an addictive drug can already modify the excitatory synaptic strengths in the VTA. Indeed, it has been extensively shown that the AMPA/NMDA ratio is increased in VTA dopamine neurons after one dose of cocaine and that some glutamate AMPA receptor 2 (GluA2)-containing AMPA receptors (AMPARs) are exchanged for GluA2-lacking ones [33, 34]. At the same time, NMDA receptor (NMDAR) function decreases. All these elements cause an impairment in eliciting long-term potentiation (LTP). Different types of synaptic plasticity in VTA dopamine neurons induced by rewarding and aversive experiences are comprehensively reviewed by Pignatelli and Bonci [35]. Midbrain dopamine neurons are central in the mesolimbic circuitry for both natural rewards and drugs of abuse [18, 36]. The VTA is known to be a central hub integrating numerous inhibitory inputs as GABAergic synapses represents 50–80% of all synapses onto VTA dopamine neurons [37–39]. GABAergic inhibition of dopamine neurons is mediated by both fast ionotropic GABA_A_ receptors and slow metabotropic GABA_B_ receptors [40].

In 2017, Edwards et al. [41] showed that the principal monosynaptic projection to VTA dopamine neurons arising from the NAc [42] inhibits the firing of dopamine neurons via activation of GABA_B_ receptors, whereas local VTA inhibitory interneurons inhibits dopamine neurons through GABA_A_ receptors. Today, it is well established that pharmacological activation of GABA_B_ receptors (e.g. by baclofen) reduces cue-associated cocaine craving as well as reduce cocaine use in humans [43–45] and it reduces rewarding and reinforcing effects of cocaine on animal models [46–49]. Edwards et al. report [41] indicates that the therapeutic effects of baclofen might pass through VTA dopamine neurons’ GABA_B_ receptors. Intrathecal Baclofen is an effective and safe long-term treatment used worldwide to treat severe spasticity [50, 51]. Oral baclofen is less effective and has significant rates of side effects, like sedation, somnolence, vertigo and headache especially when prescribed off-label for drug addiction (because higher doses are commonly used) [51, 52]^.^. Indeed, contrasting results on the effect of baclofen in reducing alcohol craving [53–55] and cocaine dependence [43, 56] were probably due to different severity of alcohol dependence of the enroled patients. This is way higher dose are tested and often prescribed off-label for drug addiction [55]. Thus, self-poisoning that could lead to severe toxicity and death represents one the major concern of baclofen use in drug addiction. Therefore, baclofen should be prescribed with caution and close monitoring [52, 57].

Together with drug of abuse-induced LTP at excitatory synapses, plasticity of GABAergic inhibitory synapse in the VTA also have an impact on the firing rate of VTA neurons, at least following opioid [58] and cocaine administration [59]. Normally, NMDA activation, during excitatory LTP (induced by high-frequency stimulation), leads to the release of NO that will activate guanylate cyclase in adjacent GABAergic terminals, which in turn, leads to increase in GABA release. This presynaptic NMDA receptor-dependent GABAergic LTP heterosynaptic plasticity, is named LTP_GABA_. Nugent el al. [58] showed that opioids blocks LTP_GABA_ through a disruption of the coupling between nitric oxide (NO) and guanylate cyclase. The incapability of GABAergic synapses to potentiate after morphine or cocaine administration may promote LTP of glutamatergic synapses [58, 59]. The early loss of inhibitory control combined with potentiation of glutamatergic synapses on dopaminergic neurons might represent adaptations that increase vulnerability to addiction [58, 59]. Furthermore, GABA_A_ receptor modulators modify the addictive drugs effects [60, 61], and targeting these receptors might be seen as an effective therapeutic strategy but precluded by many side effects among which dependence itself [62–64].

In addition to the discovery of LTP_GABA_, Nugent’s group showed that morphine is also able to modulate a form of postsynaptic LTD (LTD_GABA_) at GABAergic synapses onto VTA dopamine neurons. Remarkably, after a single administration of morphine, LTD_GABA_ was absent in slices from morphine-treated rats while unaffected in slices from saline-treated rats, indicating a bidirectional control of morphine on GABAergic synaptic plasticity in the VTA [65]. This absence of LTD_GABA_ is suggested to result from an occlusion effect due to prior morphine-induced decrease in GABAergic synaptic strength through potentiation of glutamatergic transmission and mediated by endocannabinoid signalling [66]. It is also possible that morphine alters the ability of synapses to exhibit evoked LTP or LTD in the VTA. Previous experiences such as exposure to drugs of abuse, stress, visual or sensory deprivation can change the ability of synapses to undergo subsequent plasticity in response to LTP and LTD induction protocols. This concept of modification of plasticity capability is referred as metaplasticity [67].

In the NAc, chronic exposure to addictive drugs induces specific synaptic changes that are different from those of the VTA, including a decrease of the AMPA/NMDA ratio as some AMPARs are endocytosed. This leads to a depressed synapse (sometimes referred as long-term depression (LTD) like state), where NMDAR-dependent LTD is reduced or, in some experiments, abolished [68, 69]. Highlighting the importance of temporal aspects, studies of withdrawal period after chronic administration of cocaine, showed that synaptic AMPAR levels increase during the first week of withdrawal and persist elevated for weeks [70–72]. It is established that cocaine challenge transiently decreases AMPAR surface expression, while AMPARs recover back to upregulated levels within a week, with a continuous increase during what is known to be the incubation of craving stage [73].

**Fig. 3:**
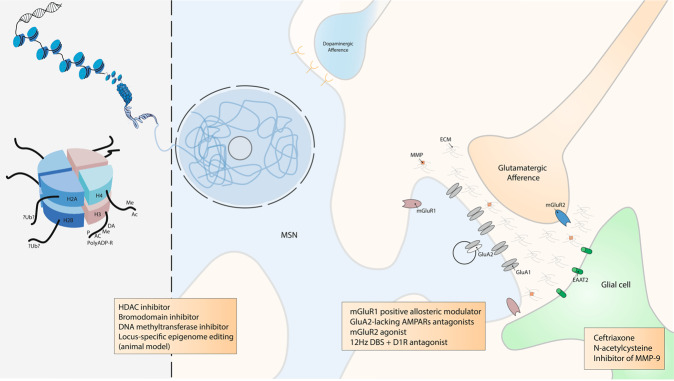
Scheme of epigenetic and tetrapartite synaptic drug-induced modifications seen as potential target of promising therapeutics. DNA is packaged inside nuclei with the help of histones. These are positively charged proteins that strongly adhere to negatively charged DNA and form complexes called nucleosomes. Each nucleosome is composed of DNA wound around histone octomers (H2A, H2B, H3 and H4). Nucleosomes fold up to form chromatin fibre, which forms loops compressed and folded to produce fibres, which are coiled into the chromatid of a chromosome. Only by loosening compacted chromatin, the DNA of a specific gene can be made accessible to transcription. Some of these drug-induced modifications at the chromatin level are extremely stable and sustain the drug of abuse-induced long-term behaviours. Among them, histone post-translational modifications (PTMs) are known to be causally involved in drug-induced behaviours [194]. PTMs include acetylation (Ac), methylation (Me), phosphorylation (P), ADP ribosylation (PolyADP-R) and dopaminylation (DA), among a growing list of newly discovered modifications [162, 172]. For example, while ubiquitylation (Ub) of H2A is known to be a key interactor of H3 methylation [253], its supposed role in drug addiction is still unknown. At this epigenetic level, some drugs were demonstrated to have an influence on drug-induces behaviours such as histone deacetylase (HDAC), bromodomain and DNA methyltransferase inhibitors. Locus-specific epigenome editing is now encouraging as a new field of investigation as it might help to the discovery of new specific and causal drug of abuse targets. Overview of the tetrapartite glutamatergic synapse composed of a medium spiny neuron (MSN), a glutamatergic projection, a glial cell and the extracellular matrix (ECM). Here, we focused on synaptic potentiation after drug of abuse administration with the addition at the post-synaptic membrane of glutamate AMPA receptor 2 (GluA2) lacking AMPA receptors (AMPARs). This mechanism might be reduced by metabotropic glutamate receptor 1 (mGluR1) positive allosteric modulator or more directly by GluA2-lacking AMPARs antagonists. In the same way, it was also shown that presynaptic mGluR2 agonists can potentially abolish drug seeking and impair craving incubation. Optogenetically-inspired 12 Hz deep brain stimulation (DBS) in the nucleus accumbens can also be a promising novel therapeutic for addiction. Finally, ceftriaxone, N-acetylcysteine, and inhibitor of matrix metalloproteases 9 (MMP-9), mainly through their action on glial cell and the ECM, are very interesting molecules that may be added in the addiction therapeutic arsenal.

The abstinence period after withdrawal is of particular interest considering the classical progression of the disease, the chance of relapse and the opportunity for new therapeutic targets. A seemingly counterintuitive concept named ‘incubation of cocaine craving’ was introduced by Grimm et al. [74] who modelled cocaine-craving behaviour by using rats trained to press a lever to receive an injection of cocaine and were then forced in a withdrawal period where cocaine reward was no longer given. This concept of ‘incubation’ did not originate in drug addiction research but came from a four-stage model of the creative process proposed by Graham Wallas in 1926 [75]. Consistent with clinical observations in humans [76–78], they showed that relapse was progressively stronger over 2 months of cocaine withdrawal and suggest that a craving syndrome progresses or ‘incubates’ during the first 2 months of cocaine abstinence, and probably lasts for longer [74]. Subsequently, it was shown that this increase was due to the addition of new AMPARs lacking GluA2 and that these new receptors mediate the ‘incubation of cocaine craving’ [72]. Conrad et al. [72] showed that after extended withdrawal from cocaine, addition of synaptic AMPARs together with the increased conductance of GluA2-lacking AMPARs triggers higher sensitivity of NAc neurons to cocaine-related cues, leading to a strengthening of drug craving syndrome and relapse. In line with these results, it was suggested that as soon as abstinence is reached, the risk of relapse might be reduced if GluA2-lacking AMPARs were inactivated or removed from NAc synapses. It was thus proposed that GluA2-lacking AMPARs could be a new target for drug development for the treatment of cocaine addiction. While these calcium permeable AMPARs are also critical for the pathogenesis of numerous other neurological disorders (including epilepsy [79], fragile X syndrome [80], amyotrophic lateral sclerosis [81], Parkinson’s [82] and Alzheimer’s [83] diseases), developing drugs that specifically target them and not calcium-impermeable AMPARs, which are critical for normal CNS function, is challenging [84] (Fig. 3).

Inspired by previous work performed in the VTA showing that metabotropic glutamate receptor 1 (mGluR1) LTD induces removal of GluA2-lacking AMPARs from synapses [33, 34], Loweth et al. [85] demonstrated that synaptic GluA2-lacking AMPAR decrease could be accomplished by in vivo evoked mGluR1 LTD in the NAc. More importantly, their group showed that after prolonged cocaine or methamphetamine withdrawal, systemic injection of a mGluR1 positive allosteric modulator attenuated the expression of incubated craving by reducing GluA2-lacking AMPARs in the NAc [85, 86]. These results suggest a strategy in which abstinent methamphetamine or cocaine users could use a systemically active compound to protect themselves against cue-induced relapse.

These latter studies were conducted without differentiating between D1 receptor D1R MSNs and D2R MSNs. In 2014, Pascoli et al. [87] demonstrated that this increase in the strength of excitatory afferents was exclusively related to D1R MSNs. Interestingly, the type of drug-evoked plasticity involved is also dependent on the input. It has been shown that even in the same D1R MSN a synapse connecting the PFC to the NAc increases its strength by inserting GluA2-lacking AMPARs whereas a synapse connecting the ventral hippocampus to the NAc increases the AMPA/NMDA ratio by inserting GluA2-containing AMPARs [87].

Besides operant self-administration, all these long-term synaptic modifications also underlie behavioural changes associated with drugs of abuse, such as locomotor sensitisation [88, 89]. Locomotor sensitisation is a behavioural protocol used to model drug-induced behaviour [90, 91]. In rodents, repeated cocaine injection induces gradually increased locomotor activity; after 5 days of consecutive injections, the locomotor response reaches a ceiling level. This state lasts for months after cocaine withdrawal [91]. As an experimental model, locomotor sensitisation is linked with increased tendency to self-administer psychostimulants [92, 93] and with reinstatement of previously extinguished self-administration [94, 95]. Whereas the existence of psychomotor sensitisation in humans is discussed [96, 97], it is a key aspect of vulnerability to drug addiction and relapse, specifically drug craving or compulsive drug-seeking behaviour [91, 98, 99]. Still, locomotor sensitisation can be dissociated from the rewarding effect of a drug of abuse and conditioned place preference or self-administration are more appropriate experimental paradigms to test this aspect [100–102]. Even if drug-induced locomotor sensitisation is unclearly present in humans, as an animal model it offers a clear readout to understand the mechanisms by which drugs of abuse induce long-term brain modifications [91].

Furthermore, it has been elegantly demonstrated that optogenetic stimulation of the excitatory projections to the NAc is able to reverse cocaine and alcohol-evoked plasticity [87–89]. Briefly, applying a NMDAR or mGluR1-dependent LTD on cortico-accumbal glutamatergic synapses, before a drug of abuse administration, diminishes its effect. In another study, Luscher’s team took advantage of the knowledge, obtained from optogenetic in vivo experiment in rodents, to implement a novel deep brain stimulation (DBS) protocol that abolishes behavioural sensitization to cocaine (and thus that would be efficient during the relapse phase) [103]. Basically, the idea is to manipulate synaptic plasticity in the NAc to reverse pathological synaptic transmission and its associated behaviours following exposure to drugs of abuse. In this study, as a therapeutic use of optogenetic tools in humans is for now inapplicable [104], the authors reversed cocaine-evoked plasticity and thus drug-induced behaviours by using DBS instead of optogenetic. Indeed, DBS is routinely used in clinic and a new DBS protocol can easily be translationally implemented to the human therapeutics [105, 106]. They refined the classical high-frequency DBS protocol (that has no sustained effect on cocaine sensitization, probably because it does not affect synaptic plasticity) by applying a low frequency stimulation (12 Hz to equal the one used in the optogenetic endocannabinoid- dependent LTD protocol) in the NAc together with the administration of a D1R antagonist necessary to unmask the mGluR-dependent LTD in D1R MSNs as demonstrated previously [107] (Fig. 3, see section on clinical treatment for broader discussion on DBS).

Kalivas’ group showed in 2009 [108] that after extended withdrawal from chronic cocaine self-administration, cocaine-induced metaplasticity at the excitatory synapses in the NAc that impairs the ability of PFC stimulation to produce LTP or LTD in NAc MSNs. They also showed that N-acetylcysteine reverses cocaine-induced metaplasticity, allowing the induction of both LTP and LTD and that N-acetylcysteine decreases cocaine-relapse in a rodent model. We are currently awaiting the results of a randomised and control study that is testing newly detoxified (and therefore abstinent) hospitalised patients who received a 3–4 week course of treatment, in order determine if N-acetylcysteine can be a useful medication candidate to avoid relapse in patients with cocaine dependence (NCT03423667).

GABAergic D1R and D2R MSNs, equally compose and are mosaically intermingled throughout the striatum [109]. As explained above, D1R and D2R MSNs send axonal projections outside the striatum, forming the two main output pathways, respectively the direct and indirect pathways [16, 17]. In a certainly oversimplified model, the activation of the D1R MSNs result in facilitation of locomotion, reward, and reinforcement while the activation of D2R MSNs result in opposing effects [110–113]. In addition to the long-range projections, these neurons form short-range synaptic connections with one another within the striatum, and because they consist of inhibitory collaterals, a mechanism known as lateral inhibition [114–117]. Interestingly, these connections are not symmetrical, with D2R MSNs forming more synaptic connections on D1R MSNs [115, 117]. Through this previously understudied collateral transmission, Dobbs et al. [115] presented a novel mechanism by which cocaine exerts its stimulant effect: cocaine, by blocking DAT receptors enhance levels of dopamine and subsequently activating D2Rs, causes a suppression of lateral inhibition and thus disinhibition of D1R MSNs in the NAc which in turn promotes locomotion [115]. Furthermore, Alvarez’ group suggested that constitutive low D2R levels, through imbalanced lateral inhibition, might pre-sensitised D1R MSNs, facilitate behavioural plasticity to repeated cocaine and promotes an addiction vulnerable phenotype [116].

The characterisation of the role of glia and the extracellular matrix (ECM) in drug-induced synaptic plasticity is an exciting emerging field of drug addiction research as it comes with promising new therapeutic possibilitiess [118–120]. Mulholland et al. [118] summarised and emphasised the role of the ECM and of astroglial cells in the regulation of synaptic plasticity. Of great interest, restoring downregulated glutamate transporter 1 (EAAT2) with ceftriaxone reduces drug seeking in animal models [121, 122]. Matrix metalloproteases (MMP) are important regulators of the ECM and contribute to synaptic plasticity [123]. Inhibiting their activity result in suppression of the reinstatement of cocaine conditioned place preference [124] and selectively inhibiting MMP-9 prevents cue- and cocaine-induced reinstatement of cocaine self-administration [119]; these results open additional therapeutic possibilities with the use of inhibitors of MMP-9 as an innovative targeted approach [119, 124, 125] (Fig. 3). Still, at our knowledge, there are no randomised controlled study currently investigating these ECM-related drugs.

Drugs of abuse-induced modifications in glutamatergic nuclei targeting the NAc, or the VTA and essential part of the reward circuit, are less studied than cortico-striatal synapses despite the fact that they play a crucial role in the development of drug addiction. Indeed, in the OFC and PFC, chronic alcohol exposure significantly increases LTP in pyramidal neurons [126, 127]. Kazanetz et al. [128] showed that repeated cocaine injections impair endocannabinoid-LTD and mGluR2/3-LTD in the PFC. They postulated that this might mechanistically participate in the induction of a postsynaptic, observed LTP-like phenomenon with an enhanced AMPA/NMDA ratio. It was also demonstrated that neurons of the infralimbic cortex present a decrease in mGluR2 [129]. In addition, alcohol-dependent rats exhibit an escalation of ethanol seeking, which was abolished by restoring mGluR2 expression in the infralimbic cortex via viral-mediated gene transfer [129]. Notably, mGluR2 agonist was shown to impair the incubation of cocaine craving [130] and to attenuate reinstatement of cocaine-seeking [131, 132](Fig. 3). Recently, Caprioli et al. [133] extensively reviewed preclinical studies on allosteric modulators of mGluRs on animal models of drug addiction and their potential translational implications. The results reviewed [133] indicate an remarkable effect of allosteric modulators of presynaptic mGluR2 and possibly mGluR7, supporting the idea that these compounds should be tested as potential medications for addiction treatments.

Besides the PFC, other brain regions appear to be key areas in drug addiction as the paraventricular thalamus (PVT) - a central hub for cortical, sensory and limbic information [134–140]. In 2016, Zhu et al. [141] showed that chronic morphine administration potentiates excitatory synapses between the PVT and D2R MSNs via insertion of GluA2-lacking AMPARs. Remarkably, in vivo optogenetic depotentiation at these synapses abolishes morphine withdrawal symptoms. In a recent paper, projections from the PVT to the NAc were shown to be critical for augmentation of heroin seeking in food-restricted rats [142] (Fig. 1). Actually, Otis et al. [143] demonstrated that the PVT is an integrative hub for reward seeking behaviour and that PVT-NAc neurons integrate different inputs from the PFC and the lateral hypothalamus to precisely guide reward seeking behaviour. In a recent review, De Groote et al. [140] focused on the new advances in the understanding of the roles of the PVT-NAc connections in motivated behaviours, highlighting their implications in drug addiction.

### Drug addiction-related genes and transcriptomic regulation

Modifications in gene expression contribute to the long-lasting effect sustaining drug addiction; thanks to gene-expression arrays, RNA-sequencing and candidate gene approaches, the specific genes and their regulatory transcriptomic mechanisms involved in drug addiction development and maintenance are now better understood.

### Drug addiction-related genes

For example, the use of conditional gene knockout in mice emphasises the importance of monoamine membrane transporters (dopamine transporter, and serotonin transporter) [144, 145] and of mGluRs [146, 147]. As new animal models of drug addiction, these approaches are also useful to better characterise fine-tuning of important pathways involved in addiction. For example, a scaffold protein known as Maged1 has been shown to be involved in cocaine reward and reinforcement [148]. We demonstrated that Maged1 inactivation impairs drug-evoked dopamine release and glutamatergic synaptic plasticity in the NAc. Inactivation of Maged1 in mice was able to abolish behavioural sensitization to cocaine as well as cocaine conditioned place preference and operant self-administration behaviours [148]. This sole genetic alteration, causally linked to a strong alteration of drug-induced behaviours, impairs (at least) two core neuronal mechanisms leading to addictive behaviours: (1) cocaine-evoked release of dopamine in the NAc and (2) NAc plasticity, with a reduced AMPA/NMDA ratio and a resistance to LTD. Actually, it seems that, after Maged1 inactivation, the excitatory synapses in the NAc shift to a depressed state. Our hypothesis is that, in line with the previously discussed in vivo optogenetic induced LTD, this impairment could be a key factor for the significant decrease in sensitization to psychostimulants [87, 103, 148]. Actually, it seems that placing neurons in a state of ‘presensitization’ is able to prevent drug-induced sensitization itself [148, 149]. Our group is now trying to understand what are the cellular and molecular pathways directly altered by Maged1 inactivation and responsible for this strong anti-addictive drug phenotype. Remarkably, the promoter of Maged1 was found in a list of 213 promoters that co-precipitate with acetylated histones and with the activated form of cAMP response element binding protein (CREB) after chronic drug taking [150]. In line with this result, preliminary and unpublished results from our laboratory point out a specific epigenetic mechanism, in parallel with an alteration of synaptic plasticity in excitatory projection to the NAc, that would link Maged1 to its major effect on drug-induced behaviours. This selected gene approach is of great interest in refining our knowledge of pathways hijacked by addictive drugs. Using cell sorting of D1R MSNs and D2R MSNs as described previously [151], our group also identified the G-protein-regulated inducer of neurite outgrowth 3 (GPRIN3) in both MSN populations but strikingly more expressed in D2R MSNs [149]. The GPRIN family (GPRIN1, GPRIN2 and GPRIN3) are Gαi/o-regulated proteins suggested to intermediate the communication between GPCRs and the sequential intracellular target [152]. Indeed, GPRIN1 and GPRIN2 have been described as alternative (to adenylyl cyclase) mediators of GPCRs signalling but GPRIN3 had a much less defined role [152, 153]. To understand the role of GPRIN3 in the pathophysiology of the D2R-indirect pathway, we induced a D2R-MSNs-specific knockdown (KD) of GPRIN3 using small hairpin RNA and lentiviruses [151, 154]. We first observed a significant increase in distal branching, the points of convergence between glutamate and dopamine synapses in MSNs [155] and also key targets of cocaine, which itself promotes increase in distal branching in the NAc of mice [156–159]. Thus, we tested the cocaine acute effect and locomotor sensitization and observed a decrease in cocaine-induced hyperlocomotion after inactivation of GPRIN3 using a CRISP/Cas9 approach. The significant increase in distal branching in GPRIN3 D2R-MSNs KD corroborates our hypothesis that the lack of GPRIN3 induces a ‘presensitization process’, able to change the targets of cocaine and therefore altering its effects [149]. Finally, we provide the first evidence that GPRIN3 partners with D2R in the striatum and modulates cocaine-induced behaviours [149].

### Transcriptomic and epigenetic regulations

Epigenetics is a broad field and has multiple definitions that comprise several biochemical mechanisms (including DNA methylation and histone modifications) sustaining modifications in gene expression throughout the lifecycle of an organism without mutations of the DNA itself [160–162]. Epigenetics can be considered as the process through which environment (and normal development) interacts with an individual’s genome to determine all phenotypic traits, in health and disease. Stable modifications in gene expression are also said to be ‘epigenetic’, because they are heritable in the short term (through mitosis) [160] and in some cases trans-generationally, thus, providing a potential mechanism for environmental influences to be passed from parents to offspring [163–165]. Handel and Romagopalan [163] mentioned that “epigenetics allows the peaceful co-existence of Darwinian and Lamarckian evolution”. Such trans-generational epigenetic inheritance of drug addiction vulnerability remains debatable [161], but has been increasingly studied for the last 20 years [166, 167]. Some epigenetic changes are very stable, an thus mediate both drug addiction susceptibility and drug-induced brain alterations that underlie the development of drug addiction [161].

As the NAc is seen as the central hub of drug addiction, with the notion that chronic drug use induces long-lasting structural, electrophysiological and transcriptional changes in the NAc, researchers are mostly targeting epigenetic modifications in NAc cells. Still, considering initial reports of cocaine-induced epigenetic modifications [168, 169], it might be relevant to study further epigenetic changes in other regions such as glutamatergic inputs to the NAc, and further in the VTA, as they are implicated in the physiopathology of drug addiction [170, 171] as mentioned above.

To date, the three main epigenetic mechanisms consist of (1) DNA methylation, (2) action of the non-coding RNAs and (3) histone post-translational modifications (PTMs). As an illustrative example, we will focus here only on histone PTMs. PTMs of histone residues on their N-terminal tails, that protrude from the nucleosome core, control chromatin condensation and the switch between euchromatin and heterochromatin and thus DNA-accessibility and gene expressions. PTMs include acetylation, methylation, phosphorylation, ADP ribosylation, ubiquitylation and sumoylation, among a growing list of newly discovered modifications [162, 172].

Among these PTMs, the most studied is the acetylation of H3 and H4, that is increased in the NAc after chronic exposure to drugs of abuse [150, 173, 174]. This increase in global acetylation levels is the result of drug-induced alterations in the balance of histone acetyltransferase and histone deacetylase (HDAC) function and is associated with gene activation. CREB-binding protein, a histone acetyltransferase critical to memory processes [175], is required for cocaine-induced increases in histone acetylation in the NAc [176].

Fifteen years ago, Tsankova et al. [177] showed that imipramine, a monoamine reuptake inhibitor used for decades to treat depression, was effective through histone remodelling in depression and highlight the therapeutic potential for chromatin regulation with histone methylation and deacetylation inhibitors in depression. Nevertheless, like with synaptic plasticity (see above), discovering a drug that would interfere with epigenetic mechanisms and thus decrease drugs of abuse effect faces temporal aspects issues [173, 176, 178–182]. Indeed, timing has a strong impact considering conflicting results obtained after experimental manipulations of histone acetylation. An acute administration of HDAC inhibitors systemically or directly into the NAc, promotes behavioural responses to the drugs. However, prolonged administration decreases cocaine behavioural effects. In 2013, adding a new layer of complexity, Kennedy et al. provided comprehension to this time-dependent regulation [183]. Remarkably, they showed that prolonged intraNAc administration (but not acute administration) of a HDAC inhibitor attenuated cocaine behavioural effects by inducing a form of repressive histone methylation. This study showed, for the first time, cross-talk among different types of histone modifications [183]. Besides cross-talk between different epigenetic modifications, multiple modifications work in parallel and there is often a decoupling between an observed modification at a specific locus and its final transcription [161]. Decoding these chromatin marks will be a future challenging field. Like with HDAC inhibitors, there are promising findings based on the use of DNA methyltransferases inhibitor [184, 185] (Fig. 3). Though, the main issue with these new potential treatments for drug addiction is their lack of specificity. One of the key challenge for the pharmaceutical industry will be to generate small molecules with more specific targets [6].

While histone acetylation and methylation are increasingly studied, an important field of future investigation will be to understand the other drug-induced histone PTMs. It already seems that chronic cocaine alters levels of histone phosphorylation [174, 186, 187], and poly-ADP ribosylation [188]. Recently, an unexpected role for the intracellular dopamine in VTA has been revealed, showing that DA interacts with chromatin to initiate a new form of epigenetic regulation called dopaminylation [189] (see Table 1 for a summary of cocaine-related epigenetic modifications).

**Table 1:** Example of major chromatin modifications and their known effects on transcription.

Epigenetic modification		Localisation	Effect on gene expression	Global effect of chronic cocaine in the NAc	Refs.
Histone PTMs	Acetylation	H3	Activation	Increase	Kumar et al. [178]; Renthal et al. [150]
		H4	Activation	Increase	Brami-Cherrier [174]; Kumar et al. [178]; Renthal et al. [150]
	Methylation	H3k9me3	Repression	Decrease	Maze et al. [191]
		H3K9/27me2	Repression	Increase	Renthal et al. [150]
		H3K27me3	Repression	Balance	Feng et al. [194]; Maze et al. [201]
	Phosphorylation	H3pS10	Activation	Increase (not in D2)	Bertran-Gonzalez et al. [186]; Brami-Cherrier [174]; Jordi et al. [251]
	Poly-ADP ribosylation (PAR)	H1 PAR H3 PAR	Activation	Increase	Scobie et al. [188]
	Dopaminylation	H3Q5dop	unknown	Reduced	Lepack et al. [189]
DNA methylation		5-mC predominantly at CpG sites	Repression	Unknown	Nestler [161]

Global effects of chronic cocaine exposure on histone post-translational modifications (PTMs) in the nucleus accumbens (NAc) are indicated.

Further studies showed that histone PTMs that occur in the NAc after chronic drug administration are locus specific [150, 190, 191]. Even though, drugs of abuse alter global levels of multiple histone PTMs, such as increased histone acetylation or decreased methylation in the NAc, genome-wide studies have confirmed that a greater number of genomic sites show increased acetylation [150] or decreased methylation [190, 191]. Conversely, hundreds of genes show opposite or no changes in these same PTMs after drug exposure. What defines whether, and in which direction, a specific gene is modified in the context of a global histone PTM is an intriguing and unsolved question [161]. These genome-wide studies (ChIp on chip or ChIpSeq) are nowadays fundamental to understand where PTMs and other epigenetic modifications are deposited. This will be fundamental to guide new therapeutics.

Actually, with new tools such as zinc finger proteins (ZFPs) DNA-binding domains and, more recently, RNA-guided CRISPR/dCas9 (drastically easier to design) [192, 193], it is now possible to control epigenetic modifications at a single gene in a specific type of cell in a specific brain region [162]. Heller et al. demonstrated that gene-targeted epigenetic editing (targeted to the Fosb [194] and *Cdk5* [195] locus with ZFP technology) can alter drug-related behaviours [194, 195]. This represents crucial evidence that gene-specific changes to the epigenome are not simply correlated, but rather causal, in regulating transcriptional responses to drugs of abuse administrations. These new results of “causal epigenomics” are very encouraging as they open the way to precise translational therapeutic approaches for drug addiction and other CNS diseases.

### Linking epigenetics and synaptic plasticity

Today, most studies investigate synaptic plasticity and epigenetic as two distinct fields and it is not clear how these research topics are connected to each other. Understanding how epigenetics is connected to synaptic plasticity is an emerging research issue [6].

Of course, bridging epigenetic mechanisms with synaptic plasticity is not limited to drug addiction field. For example, in 2011, Monsey et al. [196] elegantly demonstrated that DNA methylation and histone H3 acetylation regulate auditory fear conditioning and its related synaptic plasticity in the amygdala. In 2014, Massart et al. [197] suggested that sleep deprivation induces epigenetic modification (alteration in DNA methylation and hydroxymethylation) that triggers synaptic plasticity modifications by changing expression of plasticity related genes.

Regarding drug addiction, some epigenetic marks seem fundamental and upstream as illustrated by HDAC inhibitors effect on drug-induced synaptic and behavioural modifications [178, 198–200]. Additionally, Maze et al. [201] demonstrated morphological plasticity induced by cocaine through the histone methyltransferase G9a. Again advocating for causal epigenetic, Authement et al. [66] demonstrated that HDAC inhibition locally in the VTA is sufficient to reverse epigenetic modifications and synaptic plasticity changes after morphine administration.

Two transcription factors implicated in addiction exemplify this bridging attempt: CREB and ∆FosB (a truncated form of the FosB gene) are both activated by several drugs of abuse [202]. CREB activation occurs in both subtypes of NAc MSNs (D1R and D2R), while ∆FosB activation is limited to D1R MSNs in response to all drugs of abuse except for opioids, which remarkably induce the protein in both MSNs [203]. Expression of active CREB in NAc MSNs increases their excitability [204] and underlies drug-induced long-term synaptic plasticity and associated changes in dendritic spine plasticity [205]. ∆FosB is also linked to synaptic plasticity but evokes contrasting effects on the two MSN subtypes, with increased AMPA receptor function induced in D1R MSNs and decreased AMPA receptor function induced in D2R MSNs [206]. Renthal et al. [150] unravelled CREB and ∆FosB target genes and observed that these genes are mainly involved in neuronal excitability and synaptic function. Moreover, as already briefly discussed above, CREB and ∆FosB action have also been related to multiple epigenetic regulations, including histone acetylation and methylation [150]. Besides, a novel mechanism for bridging the gap between epigenetic control of transcription and synapse plasticity might be seen in microRNAs [207]. The most studied miRNA in the context of synaptic plasticity is miR-132 and is known to be CREB-dependent [208]. In the striatum, miR-212 targets the epigenetic regulator methyl CpG binding protein 2 (MeCP2). MeCP2 acts as a transcriptional repressor through recruitment of histone deacetylases to methylated DNA segments [209, 210].

### Clinical treatments for drug addiction

Besides psychosocial interventions [211] such as cognitive behavioural therapy, the most widely used treatment for drug addiction involves agonist-like medication, a solution inadequately called replacement or substitution therapy [212]. This type of treatment has been successfully implemented in the daily practice for opioid use disorder (e.g.: methadone, buprenorphine) [213] and tobacco use disorder (e.g.: nicotine patch or gum, varenicline) [214]. Currently, this agonist-like treatment is also promising for psychostimulant use disorder [215]. Still, considering the addictive drug-like effect, the risk of abuse, misuse and diversion, replacement therapy should be prescribed with caution [215, 216].

Recently, a randomised and control study on a cocaine vaccine failed to show an effectiveness but instead raised an important issue: immunised subjects may have increased their cocaine use to overcome the competitive anti-cocaine antibody inhibition [217]. Even though significant improvements have been developed for immunopharmacotherapies for psychostimulant addiction over the last decade, very few candidates have been evaluated so far in clinical trials [218]. These considerations are some of the reasons why other treatments for drug addiction should emerge with the help of neurobiological research [219].

Following successful subthalamic nucleus DBS for Parkinson’s disease [220–222], DBS was investigated for diverse psychiatric diseases including depression [223], obsessive-compulsive disorder [224] and Tourette syndrome [225]. Today, indications for DBS are enlarging, with several positive case reports and small cases series that studied NAc DBS for drug addiction. The first studies showing potential positive effects on drug addiction were reports on application of NAc DBS primarily intended for other medication-refractory neuropsychiatric disorder where a comorbid drug addiction was unexpectedly resolved [226, 227]. For DBS treatment in drug addiction, it seems that clinical empirical results led to further bench investigations and refinement [88, 103, 228–231], or at least, clinical and animal studies evolved in parallel with poor connectivity between the two.

Afterwards, many case reports and small cases series studied NAc DBS being used primarily for drug addiction, all showing encouraging decreases in drug use [232–237]. However, these studies are limited by their descriptive nature, inconstant follow-up, multiple publication bias, small patient numbers and lack of blinded stimulation and standardised outcome measures. At this stage, additional preclinical and clinical research are needed to clarify the role of DBS in the treatment of drug addiction [237]. Currently, randomised and control clinical studies are conducted (NCT01245075).

In a recent review, Sanna et al. [238] highlighted how repetitive transcranial magnetic stimulation (rTMS) confirms the hypodopaminergic hypothesis of drug addiction. While enhancing dopaminergic function through direct or indirect pharmacological approaches does not significantly alleviate symptoms, in numerous studies, and has not yielded a single FDA-approved medication [239], rTMS might indirectly modulate the dopaminergic system. Many rTMS studies stimulate the dorsolateral PFC [240, 241] that projects to the VTA and thus induces an increase in dopamine release in the synaptic cleft in the NAc [55, 242, 243]. Nevertheless, considering the heterogeneity of methods used in rTMS studies during the last 10 years [238], protocols and guidelines, were recently suggested by an international network of experts in neuromodulation and addiction to improve homogeneity of studies [244]. From this report, it is clear that multiple technical details for optimal stimulation need further investigations that might be achieved through preclinical studies. For example, low frequency (but not high-frequency) rTMS before methamphetamine exposure in rats blocked drug-induced conditioned place preference [245]. Being non-invasive, with insignificant side effects, rTMS could be seen as a great opportunity for drug addiction treatment. We are currently waiting for the results of a randomised and control study that aims at determine if, in heavy alcohol users, a single session of TMS can lower a patient’s craving and brain response to alcohol cues (NCT02939313).

Interesting views of clinical treatments for drug addiction are discussed in some other reviews [212, 215, 216, 219]. The clinical impact of new treatments also depends on their translation into clinical practice which is mainly promoted by the pharmaceutical industry [219]. Indeed, even when an effective treatment is identified through basic research, it is commonly challenging to translate it to clinical practice, as illustrated by naltrexone as a treatment of alcoholism [219]. Another example of problematic translation to clinic is illustrated by modafinil, a treatment that has been reported to attenuate cocaine euphoria but for which larger clinical randomised and controlled studies showed controversial results [246, 247].

### Future directions

Drug addiction is a brain disease strongly influenced by environment and psychosocial aspects. The psychosocial conditions in which it has developed are extremely important. Exposure to conditioned cues can be a central issue in causing drug cravings and relapses, even after successful treatment, and thus they have to be minimised [2, 74, 77]. The pathophysiological aspects are particularly unsteady. For instance, as discussed in this review and in other ones [73, 248], synaptic plasticity is dynamically altered after psychostimulant administration, so that a treatment could have opposite effects depending on timing aspects of the administration protocol. In addition, a prolonged treatment may involve compensatory mechanisms, giving unexpected results (e.g.: when HDAC inhibitors and psychostimulants are both administered acutely, they have synergistic effects through hyperacetylation and thus transcriptional activation of psychostimulant-regulated target genes. Conversely, when a drug of abuse is given in the context of chronic HDAC inhibitor, compensatory mechanisms may promote acetylated histone to the promoters of genes responsible for inducing histone methylation and thus chromatin condensation and gene repression, all of which, in turn, gave opposite effect [183]). Thus, the evolution through the different stages of the disease has to be taken into account [249] and treatment must follow them. These two aspects have to be incorporated in a holistic treatment strategy. Besides, studying combination of different cutting-edge approaches, with animal models of addiction, such as targeted rTMS or DBS with more systemic epigenetic modulation might show a better restoration of altered synaptic transmission and decrease the probability of relapse in drug addiction. Basically, drug addiction is a disease that seems to be difficult to treat preventively but it is more conceivable to help patients that would be in an abstinence stage not to experience relapse of their disease. As addiction is chronic and relapsing, a good treatment outcome is a significant reduction of drug administration and long periods of withdrawal, with only sporadic relapses [2].

It is clear that the main issues for optimal therapeutic management of this specific psychiatric disease belong to its dynamic complexity, diverse temporal evolution and undeniably psychosocial aspects. In this review, we focused mostly on the effects of drugs of abuse on synaptic plasticity and epigenetic modifications. Nowadays, these two subfields are mostly studied separately and the understanding of how these two main addictive drug-induced brain modifications interact might be fundamental for addiction research [6]. Indeed, argument for clinical trials for new treatments emerge from fundamental behavioural studies that should be implemented in a global approach to the addicted patient.

## Conclusions

Here, we highlight, from a vast fundamental literature (mainly based on rodent models), promising therapeutics that would potentially treat drug addiction. Based on effect, on synaptic plasticity and epigenetic mechanisms, treatments such as GluA2-lacking AMPAR antagonists [72, 84], mGluR1 positive allosteric modulator [85], NAc 12Hz-DBS [103] (in line with other promising neuromodulation therapeutics such as rTMS or transcranial direct current stimulation [250]), N-acetylcysteine [108], HDAC inhibitors [183] or even (in very early stages of investigation) CRISPR/dCas9 epigenetic editing [194, 195] could be potential candidates for human randomised clinical trials (Fig. 3).

Finally, it is fundamental to consider the specific clinical aspects of the disease that would help to develop a personalised-treatment strategy. Indeed, after going from the bench to the bedside it will also be essential to assess the reversed route.
